# Oral progestagens before menopause and breast cancer risk

**DOI:** 10.1038/sj.bjc.6603618

**Published:** 2007-02-13

**Authors:** A Fabre, A Fournier, S Mesrine, J Desreux, A Gompel, M-C Boutron-Ruault, F Clavel-Chapelon

**Affiliations:** 1 INSERM (Institut National de la Santé et de la Recherche Médicale), ERI 20, Institut Gustave Roussy, 39, rue Camille Desmoulins, F-94805 Villejuif, Cedex, France; 2 Service de sénologie, Département universitaire de gynécologie-obstétrique, CHR Citadelle, Boulevard XIIème de Ligne, B-4000 Liège, Belgium; 3 Unité de Gynécologie Hôtel-Dieu de Paris AP.HP,Université Paris V, 1 Place du Parvis Notre-Dame, 75004 Paris, France

**Keywords:** breast cancer, progestagen, menopause

## Abstract

We examined the relationship between use of progestagen-only before menopause (except for mini-pills) after the age of 40 and invasive breast cancer risk in 73 664 women from the French E3N cohort study (mean age at start of follow-up, 51.8 years; mean duration of follow-up, 9.1 years). A total of 2390 cases of invasive breast cancer were diagnosed during follow-up. Risk estimates were calculated using the Cox proportional hazard model. Overall, ever use of progestagen before menopause was not significantly associated with risk (relative risk (RR): 1.01, 95% confidence interval: 0.93–1.11). However, we observed a significant increase in risk associated with the duration of use (*P*-value for trend: 0.012), current use of progestagens for longer than 4.5 years being significantly associated with risk (RR: 1.44, 95% confidence interval: 1.03–2.00). Prolonged use of progestagens after the age of 40 may be associated with an increased risk of breast cancer and the subject needs to be investigated further.

Breast cancer is the most frequent malignancy among women in Western Europe, North America (Ferlay *et al*, 2001) and even in Japan (Minami *et al*, 2004). The hormonal dependence of breast cancer has been clearly demonstrated (Dunn *et al*, 2005) and risk factors include early age at menarche, nulliparity, late age at first birth, late age at menopause (Rosner *et al*, 1994) and the use of oral contraceptives (OCs) (Collaborative Group on Hormonal Factors in Breast Cancer, 1996) and hormone replacement therapy (HRT) (Greiser *et al*, 2005).

Randomised trials and observational studies have strongly suggested that some synthetic progestagens, when added to an oestrogen in HRT, increase breast cancer risk more than the use of oestrogen alone (Chlebowski *et al*, 2003; Fournier *et al*, 2005; Stefanick *et al*, 2006). However, data on the impact of the premenopausal use of progestagens on breast cancer risk are limited.

As progestagens alone (i.e. not associated with oestrogen) have long been prescribed in France to premenopausal women for menstrual disorders, oral contraception, benign uterine and ovarian diseases and certain benign breast diseases (Lowy and Weisz, 2005), we have investigated breast cancer risk in relation to the use of progestagens before menopause in women after the age of 40 from the E3N cohort.

## MATERIALS AND METHODS

E3N is a French prospective cohort set up in 1990 to investigate cancer risk factors in women. A total of 98 995 women, aged 40–64 years, belonging to the MGEN, a French health insurance scheme primarily covering teachers, and residing in France agreed to be volunteer by filling in the first questionnaire and a consent form. Since June 1990, participants have been asked at approximately 2-year intervals to complete self-administered questionnaires requesting information on various exposures and medical diagnoses. Information on lifetime use of hormonal treatments, including progestagens, was first recorded in the January 1992 questionnaire. To facilitate accurate recall, a booklet presenting an extensive list and colour photographs of the hormonal treatments marketed in France was mailed to all study participants. Brand name, age at first use and duration of use were recorded for up to 24 periods of treatment. Information on hormonal treatment use was updated in each of the subsequent questionnaires. Information on the dose and the number of treatment days in the cycle was not requested.

For the present study, the progestagens on which we focused were oral progestagens prescribed alone before menopause and after the age of 40 years. In France, progestagens are mainly prescribed for gynaecological disorders such as breast pain, uterine or ovarian pathologies and irregular menstruations, for perimenopausal disorders and for contraception. ‘Mini-pills’, because they were only occasionally used in our study population, were classified as OCs and were excluded from the present analysis.

Cases were identified from self-reports of participants: all questionnaires asked them whether any cancer had been diagnosed, requesting the address of their physicians and permission to contact them to obtain the pathology reports.

For the present study, follow-up started at the date of return of the second questionnaire (sent out in January 1992). It continued until the return of the follow-up questionnaire sent out in June 1993, January 1995, April 1997, June 2000 or July 2002, whichever was answered last. Person-years accrued until that date, or until diagnosis of cancer or death, whichever occurred first.

Information on date of menopause, type of menopause, date of last menstruation, date of start of menopausal symptoms and date of hysterectomy were updated on receipt of each new questionnaire. Women for whom age at menopause could not be determined (e.g. women who reported a hysterectomy but gave no information on oophorectomy or menopausal symptoms, or women who indicated they were postmenopausal without any other information) were considered as menopausal at age 47 if menopause was artificial, and at age 51 otherwise, ages that corresponded in our cohort to the median age at menopause when artificial and natural, respectively.

Women who had a prevalent cancer other than basal-cell carcinoma before inclusion (*n*=11 200) were excluded, as well as women who had never menstruated (*n*=25), those who never reached the second questionnaire (*n*=1066) and those who did not report either their date at the start of progestagen treatment or duration (*n*=5998). To focus the study on intake of progestagen during the perimenopause period, the analysis was restricted to women who had never used a progestagen before the age of 40 and who reached menopause after the age of 40. This left us with 73 664 women for the analysis, accruing 668 033 person-years, with an average age at start of follow-up of 51.78 years (standard deviation (s.d.): 6.8) and a mean follow-up time of 9.07 years (s.d.: 2.4).

Relative risks (RR) for breast cancer were estimated using Cox proportional hazards models. Age was used as the timescale. Known risk factors for breast cancer were included in the model, as well as confounding variables if they improved model fit by the *P*<0.3 criterion; these are indicated in the footnotes of the tables. Imputation to the mode was used for adjustment factors with 5% or less of missing values. Progestagen use was included in the model as a time-dependent variable. The referent group in each model consisted of women who indicated that they had never used any progestagen alone before menopause.

Relative risks are given with 95% confidence intervals. The *P*-values for assessing possible heterogeneity in effect estimates were computed from likelihood ratio tests. The *P*-values for assessing possible trends were computed from likelihood ratio tests on continuous variables. All analyses were performed using SAS® system, version 9.1.

## RESULTS

The main characteristics of the 73 664 women included in the analysis according to use of progestagen treatment alone are shown in Table 1. At the end of follow-up, ever users had later menopause and more frequently had a personal history of benign disease of the breast, uterus or ovary than never users. Ever use of OCs (oestrogen–progestagen and mini-pills) or of HRT, and mammographic follow-up were more frequent in ever users of progestagens, and young generations were more likely to have used progestagens than older women.

During follow-up, 2390 cases of new primary invasive breast cancer were identified among the 73 664 women in the cohort. Pathology reports were obtained for 95.27% of cases. In all, 443 802 person-years were associated with never-use and 224 231 person-years with ever-use, in which 1510 and 880 cases of invasive breast cancer were recorded, respectively. Overall, there was no significant association between ever-use of progestagen and breast cancer risk (RR: 1.01; *P*=0.77). The relationship between ever-use of progestagens and breast cancer risk did not vary significantly by previous use of OC (*P* for interaction: 0.57), by personal history of benign breast disease (*P* for interaction: 0.86), by personal history of benign uterine or ovarian disease (*P* for interaction: 0.19) or mammographic history (*P* for interaction: 0.23).

However, we found a significant increase in breast cancer risk with increasing duration of use (*P* for trend=0.012, Table 2). We investigated associations according to time since first use and time since last use but did not find any significant association or trend (Table 2).

Results on duration of use were further stratified according to recency of use (Table 3). We found that, among current users, use longer than 4.5 years was significantly associated with breast cancer risk (RR=1.44; *P*=0.034), but not use shorter than 4.5 years. After discontinuation, and whatever the duration, the risks were close to unity. There was no significant trend towards decreasing risk with increasing time since last use (Table 3).

Because many women (48.4% of progestagens users) changed or temporarily interrupted their treatment, we verified that this did not modify risk patterns. Analyses conducted separately on women who had never interrupted nor changed their treatment and on women who declared at least one temporary interruption or a change yielded comparable results (data not shown).

Finally, we investigated associations according to whether treatment was possibly antigonadotrophic (cyproterone acetate, medroxyprogesterone acetate, nomegestrol acetate, chlormadinone acetate, ethynodiol, norethisterone acetate, lynestrenol and promegestone) or not (progesterone, retroprogesterone, medrogestone and demegestone). Overall, patterns of risk did not show marked differences (*P* for homogeneity: 0.35).

## DISCUSSION

We did not find a significant association between breast cancer risk and ever-use of a progestagen before menopause. However, we found a significant trend towards increasing risk with increasing duration of use, and current use of treatment for longer than 4.5 years was positively and significantly associated with risk.

A previous study showed a significant decrease in breast cancer risk associated with the use of an oral nonsteroid progestin alone (Plu-Bureau *et al*, 1994), although based on only 15 cases in a cohort of 1150 women with benign breast disease, meaning that any conclusion on the impact of progestins in the general population was difficult to draw.

Some studies found an increase in risk associated with the use of a progestagen-only HRT (Magnusson *et al*, 1999; Newcomb *et al*, 2002; Beral, 2003; Dinger *et al*, 2006), but these studies involved small numbers of cases. Also, the unusual use of a progestagen-only HRT may reflect the particular profile of the women receiving such treatment, or even misclassification (underreporting oestrogen).

Studies on HRT have shown that a combination of oestrogen plus progestin increases breast cancer risk more than oestrogen alone (Chlebowski *et al*, 2003; Fournier *et al*, 2005; Stefanick *et al*, 2006), but these involve administration to postmenopausal women.

*In vivo* studies have supported a role for progesterone in the induction of cyclic proliferation in the breast (reviewed in Graham and Clarke, 1997), although they were not consistent with clinical trials that found that percutaneous progesterone acts as an inhibitor of oestrogen-induced proliferation (Chang *et al*, 1995; Foidart *et al*, 1998). *In vitro* studies have also produced inconsistent results with progesterone acting as a proliferative (Edery *et al*, 1984; McGrath *et al*, 1985) or an antiproliferative (Clark and Peck, 1979; McManus and Welsch, 1984; Malet *et al*, 2000) agent in normal breast cells.

Overall, these results tend to suggest a deleterious effect of oral progestagens on breast cancer risk. However, different progestagens may affect risk differently, and the estrogenic environment may also modify their effect (Pasqualini *et al*, 1998), so it would be premature to conclude an overall class effect of progestagens, particularly as studies like ours specifically addressing the relationship between oral progestagens given alone before menopause (except for mini pills) and risk are rare.

Our results are consistent with a promoting effect of progestagens on tumour cells, by showing an increase in risk with increasing duration, and suggesting a return to baseline risk after discontinuation. A similar effect has been demonstrated for depot medroxyprogesterone acetate (Skegg *et al*, 1995) and for mini-pills (Collaborative Group on Hormonal Factors in Breast Cancer, 1996; Kumle *et al*, 2002); those authors suggested that recent use was positively and significantly associated with breast cancer risk, and that the risk was close to unity after discontinuation. Our results are in agreement with others (Collaborative Group on Hormonal Factors in Breast Cancer, 1996, 1997; Lee *et al*, 2005) suggesting that the increase in risk might be limited to current use of hormonal treatments.

Our study had some limitations. We did not record any details on the treatment (number of days per month, dose) and hence could not analyse the risk associated with intermittent or continuous use or with dose. Although the reasons for prescribing progestagens were not recorded, a potential ‘prescription’ bias is unlikely, because we adjusted for the variables ‘personal history of benign breast disease’ and ‘personal history of benign uterine or ovarian disease’, and because the effect of progestagens on breast cancer risk did not vary significantly by personal history of benign breast disease or by personal history of benign uterine or ovarian disease. The effect of progestagens on breast cancer risk did not differ significantly according to OC use. Misclassification of progestagen exposure, which was based on self-reported information, may have affected our results, but given the prospective design, this should be non-differential between cases and non-cases, and would tend to reduce the magnitude of the relationship with risk, and dampen differences in the effects of different progestagens.

Finally, there is limited scope for ‘surveillance bias’ owing to progestagen users being more likely to have repeated mammograms, because this was adjusted for, and because the effect of progestagens on risk did not differ significantly according to mammographic history.

In conclusion, our study suggests that the use, before menopause, of oral progestagens (without oestrogens) by women over 40 may increase breast cancer risk. Further follow-up study will enable more exhaustive analysis using specific categories of progestagens.

## Figures and Tables

**Table 1:** Selected characteristics (at the end of follow-up) of participants according to ever use of progestagens (*n*=73 664) E3N cohort study (1990–2002)

	**Non-users *n*=45 294**	**Users *n*=28 370**
	***n* (%)**	***n* (%)**
*Year of birth*		
1925–1937	18 402 (40.62)	5030 (17.72)
1938–1944	11 024 (24.33)	9109 (32.10)
1945–1951	15 868 (35.05)	14 231 (50.18)
		
*History of BC (first degree relatives)*		
None	40 191 (88.73)	25 027 (88.21)
1	4 640 (10.24)	3 074 (10.83)
2 and +	463 (1.03)	269 (0.96)
		
*History of BC (second degree relatives)*		
No	38 717 (85.47)	23 755 (83.73)
Yes	6577 (14.53)	4615 (16.27)
		
*History of BUOD* a		
No	27 199 (60.04)	14 096 (49.68)
Yes	18 095 (39.96)	14 274 (50.32)
		
*History of BBD* a		
No	35 122 (77.54)	18 418 (64.92)
Yes	10 172 (22.46)	9 952 (35.08)
		
*Body mass index (premenopausal, kg m*^−*2*^)		
<22	279 (0.61)	148 (0.52)
22–25	43 451 (95.93)	27 257 (96.07)
25–30	1214 (2.68)	772 (2.72)
>30	350 (0.78)	193 (0.69)
		
*Age at menarche (years)*		
<13	20 798 (45.91)	13 369 (47.12)
13–15	22 653 (50.01)	14 134 (49.82)
>15	1843 (4.08)	867 (3.06)
		
*Parity*		
Nulliparous	5450 (12.03)	3028 (10.67)
Age at first FTP>30 num=1^b^	1 836 (4.05)	1 101 (3.88)
Age at first FTP>30 num>1	1981 (4.37)	1129 (3.97)
Age at first FTP<= 30	36 027 (79.55)	23 112 (81.48)
		
*OC use*		
Never	31 606 (69.77)	14 927 (52.61)
Ever	13 688 (30.23)	13 443 (47.39)
		
*Age at menopause*^c^ *(years)*		
<48	9871 (25.23)	4429 (18.24)
48–52	19 990 (51.09)	12 995 (53.53)
>52	9266 (23.68)	6853 (28.23)
		
*HRT use* c		
Never	14 541 (37.16)	2679 (11.04)
Ever	24 586 (62.84)	21 598 (88.96)
		
*Mammographic history*		
Never	9660 (21.32)	2775 (9.78)
Ever	35 634 (78.68)	25 595 (90.22)

a BUOD=benign uterine or ovarian disease; BBD=benign breast disease.

b FTP=Full-term pregnancy; num=number of FTP.

c Among postmenopausal women.

**Table 2:** Relative risk associated with the use of progestagens according to duration, time since first use and time since last use, compared with never use (*n*=73 664). E3N cohort study (1990–2002)

	**PY^a^**	**Cases**	**RRa,^b^**	**95% CI^a^**
Never use	443 802	1510	1.00	
Ever use	224 231	880	1.01	0.93–1.11
				
*Duration (years)* c				
<1	83 449	272	0.90	0.79–1.03
1–2.5	63 932	272	1.00	0.85–1.17
2.5–4.5	43 712	174	1.10	0.96–1.26
>4.5	33 138	162	1.13	0.96–1.33
*Trend (per year of use)*			1.03	1.01–1.06
				
*Interval since first use (years)* c				
<4	70 591	235	1.03	0.89–1.18
4–7.5	61 804	235	1.04	0.90–1.20
7.5–11.5	48 026	209	1.04	0.90–1.21
>11.5	43 810	201	0.95	0.81–1.11
*Trend (per year since first use)*			0.99	0.98–1.01
				
*Interval since last use (years)* c				
Current use	68 697	235	1.14	0.97–1.33
<3	68 108	265	0.96	0.84–1.10
3–6	40 004	175	1.07	0.91–1.26
6–9.5	25 511	111	0.97	0.80–1.19
>9.5	21 863	94	0.92	0.75–1.14
*Trend (per year since last use)*			0.99	0.98–1.01

a PY=person-year; RR=relative risk; CI=confidence interval.

b Adjusted for BMI before and after menopause (<22/22-25/25-30/>=30), menopausal status°(premenopausal/artificial menopause/natural menopause), age at menopause (<48/48–52/>52), parity and age at first FTP (nulliparous/first FTP at age < 30/first FTP at age ⩾30, num=1/first FTP at age ⩾−30, num>1), age at menarche (<13/13–15/>15), familial history of breast cancer in sisters, mother, children (no/1/more than 1), familial history of breast cancer in other relatives (yes/no), personal history of benign breast disease °(yes/no), personal history of benign uterin or ovarian disease (yes/no), use of oral contraceptive (never/current or < 5 years after stop/ > 5 years after stop), use of HRT °(No/oestrogen alone/oestrogen+progestin/oestrogen+progesterone/others) and previous mammography (yes/no) °Time-dependent variables.

c Categories correspond to quartiles.

**Table 3:** Relative risk associated with the use of progestagens according to duration and recency of use, compared with never use (*n*=73 664). E3N cohort study (1990–2002)

	**Duration of use (years)**					
	**<4.5**			**⩾4.5**		
**Recency of use**	**[PY; cases]**	**RR^a^**	**95% CI**	**[PY; cases]**	**RR^a^**	**95% CI**
Current use	[58 751;193]	1.09	0.92–1.29	[9 946;42]	1.44	1.03–2.00
Past use	[132 341;525]	0.97	0.87–1.07	[23 193;120]	1.06	0.88–1.27
Trend (per year since last use)		0.99	0.98–1.01		0.97	0.95–1.01

a Adjusted for the same covariates as in Table 2.
